# Endoscopic versus minimally invasive surgical approach for infected necrotizing pancreatitis: a systematic review and meta-analysis of randomized controlled trials

**DOI:** 10.1080/07853890.2023.2276816

**Published:** 2023-11-06

**Authors:** Penghao Tang, Kamran Ali, Hayat Khizar, Yuanzhi Ni, Zhiwen Cheng, Benfeng Xu, Zhiwen Qin, Wu Zhang

**Affiliations:** a Graduate School of Zhejiang, Chinese Medical University, Hangzhou, Zhejiang, China; b Department of Oncology, The Fourth Affiliated Hospital, International Institutes of Medicine, Zhejiang University School of Medicine, Zhejiang, China; c Graduate School of Zhejiang University School of Medicine, Hangzhou, Zhejiang, China; d Department of Hepatobiliary Pancreatic Surgery, Shulan (Hangzhou) Hospital Affiliated to Zhejiang Shuren University, Shulan International Medical College, Zhejiang, China

**Keywords:** Infected necrotizing pancreatitis, endoscopy, minimally invasive surgery, complications, necrosectomy

## Abstract

**Background/Aims:**

Acute pancreatitis is a common condition of the digestive system, but sometimes it develops into severe cases. In about 10–20% of patients, necrosis of the pancreas or its periphery occurs. Although most have aseptic necrosis, 30% of cases will develop infectious necrotizing pancreatitis. Infected necrotizing pancreatitis (INP) requires a critical treatment approach. Minimally invasive surgical approach (MIS) and endoscopy are the management methods. This meta-analysis compares the outcomes of MIS and endoscopic treatments.

**Methods:**

We searched a medical database until December 2022 to compare the results of endoscopic and MIS procedures for INP. We selected eligible randomized controlled trials (RCTs) that reported treatment complications for the meta-analysis.

**Results:**

Five RCTs comparing a total of 284 patients were included in the meta-analysis. Among them, 139 patients underwent MIS, while 145 underwent endoscopic procedures. The results showed significant differences (*p* < 0.05) in the risk ratios (RRs) for major complications (RR: 0.69, 95% confidence interval (CI): 0.49–0.97), new onset of organ failure (RR: 0.29, 95% CI: 0.11–0.82), surgical site infection (RR: 0.26, 95% CI: 0.07–0.92), fistula or perforation (RR: 0.27, 95% CI: 0.12–0.64), and pancreatic fistula (RR: 0.14, 95% CI: 0.05–0.45). The hospital stay was significantly shorter for the endoscopic group compared to the MIS group, with a mean difference of 6.74 days (95% CI: −12.94 to −0.54). There were no significant differences (*p* > 0.05) in the RR for death, bleeding, incisional hernia, percutaneous drainage, pancreatic endocrine deficiency, pancreatic exocrine deficiency, or the need for enzyme use.

**Conclusions:**

Endoscopic management of INP performs better compared to surgical treatment due to its lower complication rate and higher patient life quality.

## 1. Introduction

Acute pancreatitis is a common gastrointestinal condition that often requires hospitalization. In approximately 10–20% of patients, necrosis of the pancreas or peri-pancreas develops [1,2]. While most cases of necrosis remain sterile, around 30% of patients develop an accompanying infection. This infection can be identified by the presence of gas in the collection, positive culture results from the necrotic aspirate, long-term sepsis, or ongoing clinical deterioration [3–7]. Recent studies have shown that minimally invasive surgery (MIS) can effectively treat such conditions. Specifically, laparoscopic cystogastrostomy with internal debridement has been found to be superior to open surgical necrosectomy with internal debridement performs better than open surgical necrosectomy [8–12]. During the endoscopic drainage procedure, an internal endoprosthesis is inserted endoscopically to facilitate transluminal drainage. This technique can involve endoscopic mechanical debridement as well as the use of percutaneous drainage catheters [13]. Based on these findings, both endoscopic and minimally invasive surgical approaches offer less invasive alternatives to open surgical necrosectomy and have proven effective in treating infected necrotizing pancreatitis. Randomized clinical trials have investigated these treatments, with the endoscopic approach showing a lower incidence of major adverse events [14–16]. The positive outcomes can be attributed to reduced surgical anxiety and associated challenges, such as pancreatic fistulas, as well as the elimination of general anaesthesia and exploratory surgical procedures. If endoscopic transluminal drainage fails to significantly improve the patient’s clinical condition, an endoscopic necrosectomy can be considered as an alternative. Alternatively, a step-up technique can be employed, where drainage is performed initially.

Many published studies have small sample sizes, making it challenging to draw definitive conclusions [14,15]. Therefore, there is a pressing need for a meta-analysis that incorporates updated information. Meta-analysis is particularly valuable when evaluating treatment effectiveness based on a large sample size, which may not be feasible through individual analyses of several trials producing negative results [14]. Previous meta-analyses have included only a small number of studies and encompassed both randomized controlled trials (RCTs) and observational studies [17–19].

In this study, we conducted a meta-analysis specifically focused on RCTs to compare the surgical and endoscopic treatments for infected necrotizing pancreatitis.

## 2. Materials and methods

To accurately present this meta-analysis, we adhered to the PRISMA (Preferred Reporting Items for Systematic Reviews and Meta-Analyses) guidelines for comprehensive reporting of systematic reviews and meta-analyses [20].

### 2.1. Search strategy

In our study, we conducted a comprehensive search of medical databases, namely PubMed, Web of Science, and the Cochrane Library, to identify relevant articles pertaining to our research topic. Various search terms and combinations, such as ‘endoscopic drainage,’ ‘surgical drainage,’ ‘Minimally invasive surgery,’ and ‘necrotizing pancreatitis,’ were utilized to maximize the scope of our search. The search period extended until December 2022, and we exclusively focused on studies published in English that involved human subjects.

To ensure a robust selection of studies, two authors collaborated in the process of gathering articles that were deemed significant for inclusion in our reference list. Discrepancies or differences of opinion regarding the final list were resolved through transparent and thorough discussions.

### 2.2. Studies selection

To ensure the inclusion of relevant and reliable studies in our meta-analysis, we employed rigorous criteria during the study selection process. We established both inclusion and exclusion criteria to ensure the inclusion of high-quality studies that met our predefined standards. By implementing these criteria, we aimed to maintain the integrity and validity of our meta-analysis results.

### 2.3. Inclusion criteria

1. Only full-text RCTs were included for evaluation.
2. Studies that involved patients diagnosed with infected necrotizing pancreatitis were considered.
3. The studies compared the incidence of adverse events and mortality between surgical drainage and endoscopic drainage as therapeutic interventions.
4. Only studies published in the English language were included.
5. The studies included patients aged 18 years or older.

### 2.4. Exclusion criteria

1. Non-randomized controlled trials, including observational studies, case reports, abstracts, reviews, or letters, were excluded.
2. Studies that did not directly compare the effectiveness of surgical and endoscopic treatments for necrotizing pancreatitis were not included.
3. Studies with only one treatment arm or missing required results were excluded.
4. Studies not published English were excluded.

### 2.5. Data extraction

Following the predefined research selection criteria, two authors independently performed data extraction. The extracted information encompassed the study names, study designs, patient demographics, disease characteristics, specifics of endoscopic and surgical interventions, as well as details pertaining to the primary outcome measures. These outcome measures encompassed rates of adverse events, endocrine and exocrine pancreatic insufficiency, and length of hospital stay. To ensure consistency, various coefficients underwent a scaling process.

Each study included two distinct arms: an endoscopic arm and a surgical arm. In the event of any disagreements regarding the extracted data, the first two authors consulted with the third author to reach a consensus and resolve discrepancies.

### 2.6. Outcomes and definitions

Our primary outcome was major complications, which were defined as the sudden loss of function or failure of one or more organs in the body, such as the heart, lungs, or kidneys, leading to death or requiring intervention. Secondary outcomes encompassed death or mortality, specific components of major complications (e.g. new onset of organ failure, bleeding), endocrine pancreatic insufficiency, exocrine pancreatic insufficiency, fistula or perforation (including pancreatic fistula), incisional hernia, additional percutaneous drainage, surgical site infection, need for enzyme use, length of hospital stay, and procedure time. Given the specificity of these secondary outcomes, no further definitions were provided.

### 2.7. Statistical analysis

The statistical analysis in our study was conducted using Cochrane Review Manager Software, version 5.4.1. Risk ratios (RRs) and their corresponding 95% confidence intervals (CIs) were calculated for each outcome. The pooled RRs and CIs were determined using the Mantel-Haenszel technique of the fixed effect model. Mean differences were calculated using the continuous inverse variance approach with a fixed effect model.

To evaluate statistical heterogeneity, we employed the Cochrane x^2^ test and assessed the I^2^ statistic. The I^2^ statistic represents the proportion of total variation across studies that is attributed to heterogeneity. Values of 25–49% indicate low heterogeneity, 50–74% indicate moderate heterogeneity, and values greater than 75% indicate high heterogeneity [21]. To explore the possibility of publication bias, a funnel plot was utilized. Statistical significance was determined by a p-value less than 0.05, indicating the data to be statistically significant.

### 2.8. Risk of bias

To assess the risk of bias, we employed the Cochrane Collaboration tools [22]. Ratings of ‘low’ indicated a low risk of bias, ‘high’ indicated a high risk, and ‘some concerns’ indicated that there was insufficient data to determine the likelihood of bias. We comprehensively evaluated various aspects including randomization, the extent of missing data in the results, the timing of participant identification or recruitment, outcome measurement, the potential bias resulting from deviations from intended interventions, and outcome selection.

### 2.9. Publication bias and study effect

To assess the presence of publication bias, we employed a funnel plot based on the final results. This graphical representation allowed us to visually examine the potential asymmetry in the distribution of study outcomes.

Furthermore, a sensitivity analysis was conducted to evaluate the impact of individual studies on the overall results. This analysis involved systematically excluding studies and observing the resulting changes in the outcomes. Studies were excluded from the analysis if their inclusion had a substantial influence on the final results, thereby ensuring robustness and reliability in our findings.

## 3. Results

### 3.1. Study selection

A total of 623 articles were initially identified through medical databases and supplementary sources. After removing duplicates and irrelevant publications based on subject and title evaluation, 372 articles remained for further assessment of eligibility. During this process, abstracts, retrospective studies, single-arm studies, case reports, reviews, letters, animal studies, as well as studies with incomplete or missing outcome data were excluded.

**Figure 1.**
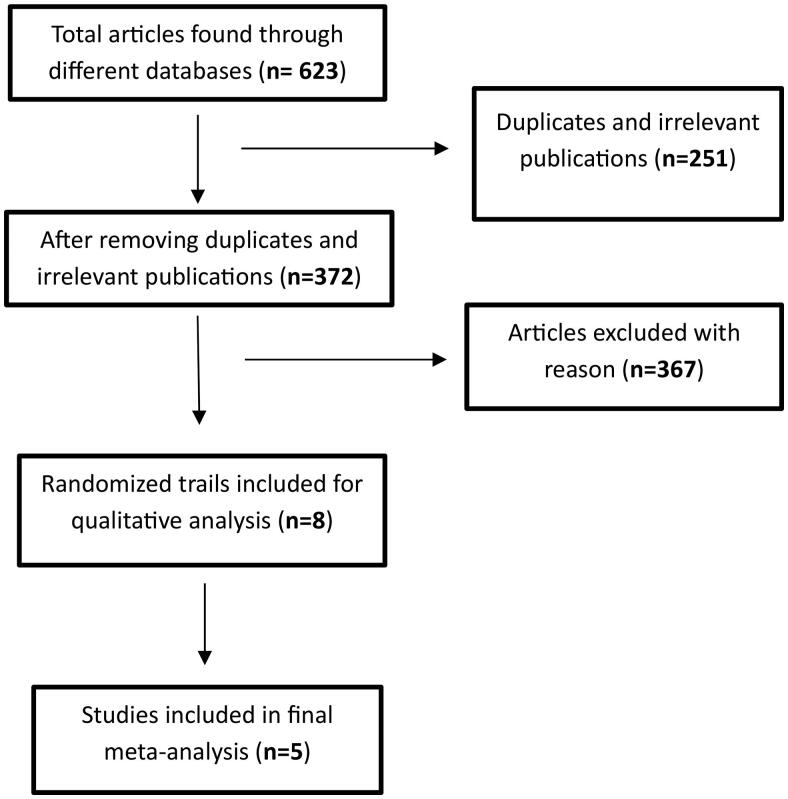
Flow chart.

As a result, a final selection of five studies met the inclusion criteria for the meta-analysis. The flow chart visually depicts the screening process and illustrates the journey from the initial pool of articles to the five articles considered in the final analysis (Figure 1 flow chart).

### 3.2. Studies characteristics

The selected randomized controlled trials investigating the management of necrotizing pancreatitis involved a total of 284 enrolled patients. Among these patients, 139 underwent minimally invasive surgical (MIS) drainage, while the remaining 145 individuals received endoscopic drainage.

These trials were conducted in different countries, including India [23,24], the United States of America [15], and the Netherlands [14,16]. Detailed characteristics of each study can be found in Tables 1–3. The aetiology of necrotizing pancreatitis varied among the studies and included factors such as alcohol consumption, gallstones, idiopathic causes, hypertriglyceridemia, post-ERCP complications, trauma, and medication-induced pancreatitis.

**Table 1.** Characteristics of included studies.

Study	Patients	Age	Male	AP ACHE 2 score	Single organ failure	Multiple organ failure	Size of collection
Bakker et al. (2012) [14]	SN = 10	64 (46–72)	8 (80)	11 (7–14)	3	1	NA
	EN = 10	62 (58–70	6 (60)	10 (6–14)	2	2	
Angadi et al. (2021) [24] RCT	SN = 20	32 (16–60)	18	NA	NA	NA	1229.4 ± 751.2
	EN = 20	36 (21–51)	17				1586.5 ± 505.2
Bang et al. 2019 [15]	SN = 32	52.9 (14.2)	21	27.1 (20.3	3	7	10.0 (3.3)
	EN = 34	55.6 (14.2)	22	33.7 (13.5)	2	7	10.0 (4.5)
Brunschot et al. (2018) [16]	SN = 47	60 (11)	29	10 (6–13)	14	7	NA
	EN = 51	63 (14)	34	9 (5–13)	13	9	
Garg et al. (2020) [20]	SN = 30	34.1 ± 12.7	22	NA	NA	NA	1166.1 ± 1086.1
	EN = 30	37.6 ± 12.9	22				1355 ± 827.9

**Table 2.** Characteristics of included studies.

Study	Major complication and death	Death	New onset of organ failure	Bleeding	Enterocutaneous fistula or perforation	Pancreatic fistula	Incisional hernia or	Use of enzyme	Percutaneous drainage
Bakker et al. (2012) [14]	8	4	5	0	2	7	NA	3	NA
	2	1	0	0	0	1		0	
Angadi et al. (2021) [24]	7	0	NA	1	1	NA	NA	0	3
	6	1		1	0			1	3
Bang et al. (2019) [15]	13	2	3	3	9	NA	2	NA	6
	4	3	2	0	0		0		5
Brunschot et al. (2018) [16]	21	6	6	10	8	13	1	13	NA
	22	9	2	11	4	2	0	16	
Garg et al. (2020) [20]	2	0	NA	NA	1	NA	NA	NA	1
	3	0			1				2

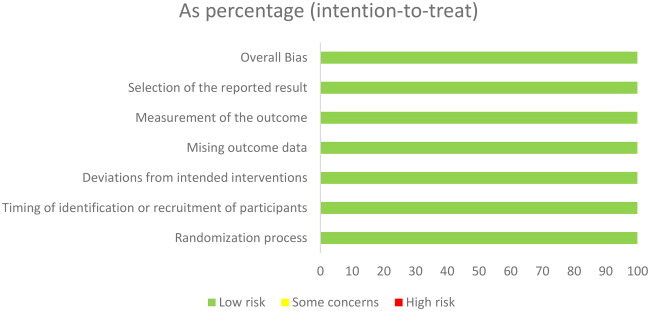

**Table 3.** Characteristics of included studies.

Study	Surgical site infection	Pancreatic endocrine insufficiency	Pancreatic exocrine Insufficiency	Length of hospital stay	Mean procedure duration	Reccurance or persistant	Need of enzyme
Bakker et al. (2012) [14]	NA	3	3	40.7 (18. 4)	NA	3	3
		2	0	42.7 (18.4)		2	0
Angadi et al. (2021) [24]	NA	NA	NA	6.5 (1.05)	101 (23)	NA	NA
				5 (1.07)	31 (19)		
Bang et al. (2019) [15]	2	9	28	23.3 (17.5)	114.6 (37.2)	5	NA
	0	6	29	16.5 (12.2)	53.5 (34.0)	4	
Brunschot et al. (2018) [16]	3	9	13	69 (38)	NA	NA	16
	2	10	16	53 (47)			13
Garg et al. (2020) [20]	5	NA	NA	NA	NA	NA	NA
	0						

Regarding the endoscopic drainage techniques, three trials utilized double-pigtail plastic stents combined with nasocystic catheters, while two studies employed a combination of lumen-apposing metal stents and double-pigtail plastic stents in EUS-guided endoscopic drainage.

Additionally, two trials utilized video-assisted retroperitoneal debridement, while three trials employed laparoscopic cystogastrostomy as the MIS approach for drainage.

### 3.3. Risk of bias and publication bias

All randomized controlled trials included in our analysis demonstrated a low risk of bias, with negligible possibility of bias in each study and outcome assessed. However, given the limited number of studies included (only 5), we did not explicitly assess the potential for publication bias.

To assess the influence of individual studies on the overall results, we conducted a sensitivity analysis by systematically excluding each study one by one. None of the studies had a significant impact on the final results, which remained consistent and unaffected by the exclusion of any specific study. However, if a study had demonstrated a substantial impact on the results, we would have excluded it from our analysis to maintain the integrity and reliability of our findings.

### 3.4. Primary outcome

#### 3.4.1. Major complications

**Figure 2.**
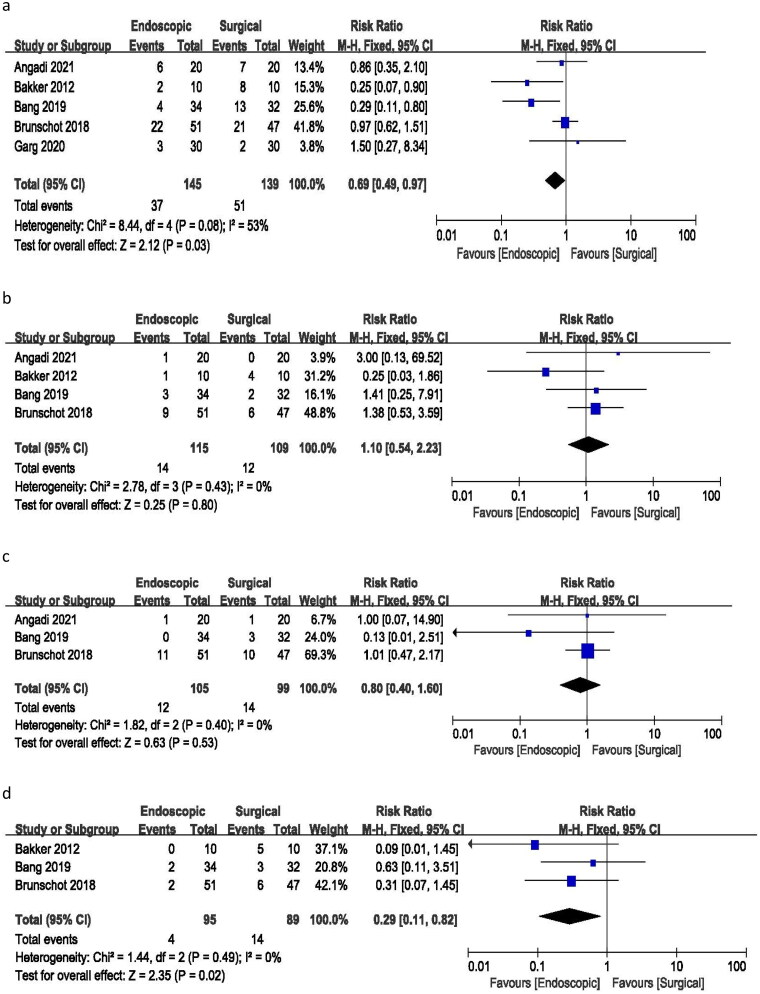
Forest plots for (a) major complications, (b) death, (c) bleeding, (d) new onset of organ failure.

All included studies provided data on major complications (sudden loss of function or failure of one or more organs in the body, such as the heart, lungs, or kidneys, leading to death). The meta-analysis yielded a risk ratio of 0.69 (95% CI 0.49–0.97), indicating a statistically significant difference in the incidence of major complications between endoscopic and surgical treatments. The analysis demonstrated moderate heterogeneity (I^2^ = 53%, *p* = 0.03) among the studies (Figure 2(a)). These findings suggest that endoscopic treatment is associated with a lower risk of major complications compared to surgical treatment.

### 3.5. Secondary outcomes

#### 3.5.1. Death or mortality

Among the five included studies, four provided data on death incidents. The meta-analysis resulted in a risk ratio of 1.10 (95% CI 0.54–2.23), with no heterogeneity (I^2^ = 0%) and a *p*-value of 0.80. These findings suggest no significant difference in the number of deaths between endoscopic and surgical treatments (Figure 2(b)).

#### 3.5.2. Bleeding

Data on bleeding incidents were available from three studies. The analysis yielded a risk ratio of 0.80 (95% CI 0.40–1.60), with no heterogeneity (I^2^ = 0%) and a p-value of 0.53. These results indicate no significant difference in the incidence of bleeding between endoscopic and surgical treatments (Figure 2(c)).

#### 3.5.3. New onset of organ failure

Three studies provided information on the incidence of new-onset organ failure. The risk ratio for new cases of organ failure was 0.29 (95% CI 0.11–0.82), with no heterogeneity (I^2^ = 0%) and a p-value of 0.02. These findings suggest a significant difference in the rate of new onset of organ failure between endoscopic and surgical procedures (Figure 2(d)).

#### 3.5.4. Surgical site infection

**Figure 3.**
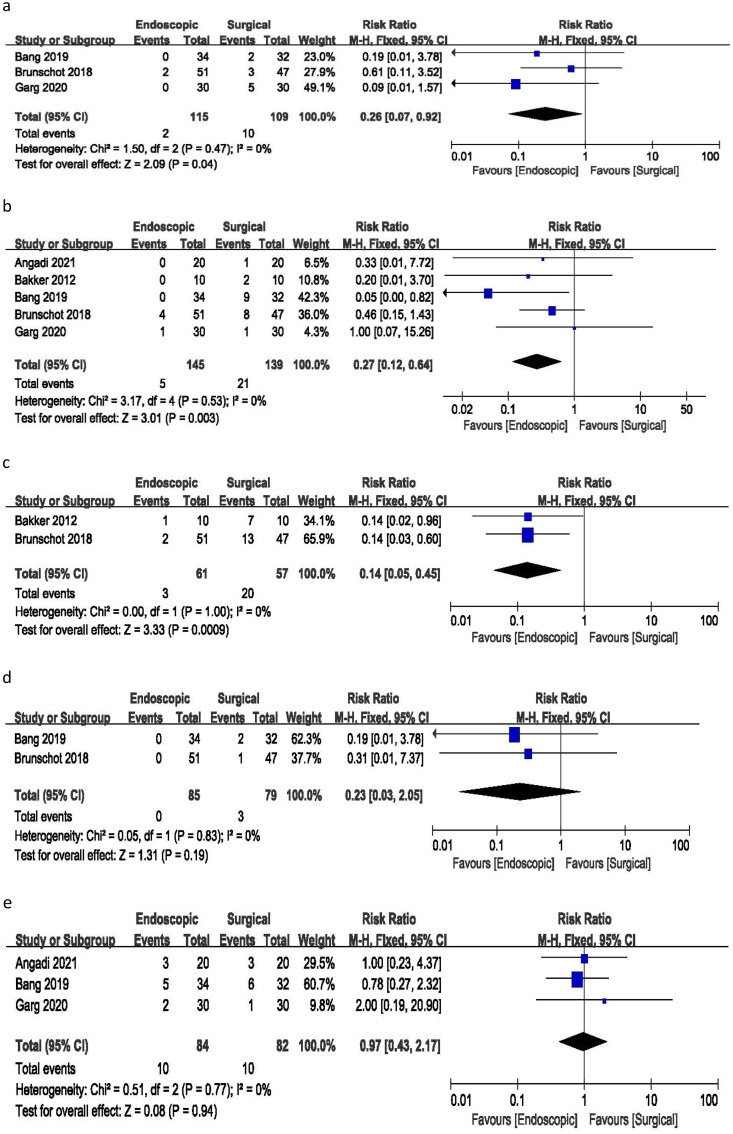
Forest plots for (a) surgical site infection, (b) fistula or perforation, (c) pancreatic fistula, (d) incisional hernia, € need for percutaneous drainage.

A risk ratio of 0.26 (95% CI 0.07–0.92) was calculated for surgical site infection based on three studies, with no heterogeneity (I^2^ = 0%) and a *p*-value of 0.04. These results indicate a significant difference in the incidence of surgical site infection between the endoscopic and surgical treatment groups, with no observed heterogeneity (Figure 3(a)).

#### 3.5.5. Fistula or perforation

All five studies included in the analysis reported incidents of fistula or perforation. The meta-analysis yielded a risk ratio of 0.27 (95% CI 0.12–0.64), with no heterogeneity (I^2^ = 0%) and a *p*-value of 0.003. These results indicate a significant difference in the incidence of fistula or perforation between endoscopic and surgical treatments (Figure 3(b)).

#### 3.5.6. Pancreatic fistula

Data on pancreatic fistula were reported in only two of the studies. The analysis resulted in a risk ratio of 0.14 (95% CI 0.05–0.45), with no heterogeneity (I^2^=0%) and a *p*-value of 0.009. These findings suggest a significant difference in the incidence of pancreatic fistula between endoscopic and surgical therapies (Figure 3(c)).

#### 3.5.7. Incisional hernia

Only two studies reported incidents of incisional hernia in the surgical group with a risk ratio of 0.23 (95% CI 0.03–2.05), no heterogeneity (I^2^ = 0%), and a *p*-value of 0.19. There was no such complication in the endoscopic group (Figure 3(d)).

#### 3.5.8. Need for percutaneous drainage

The analysis of three studies resulted in a risk ratio of 0.97 (95% CI 0.43–2.17), with no heterogeneity (I^2^ = 0%) and a *p*-value of 0.94 for the need for percutaneous drainage. These findings suggest no significant difference in the risk ratio for requiring additional percutaneous drainage between the endoscopic and surgical treatment groups (Figure 3 (e)).

#### 3.5.9. Pancreatic endocrine deficiency

**Figure 4.**
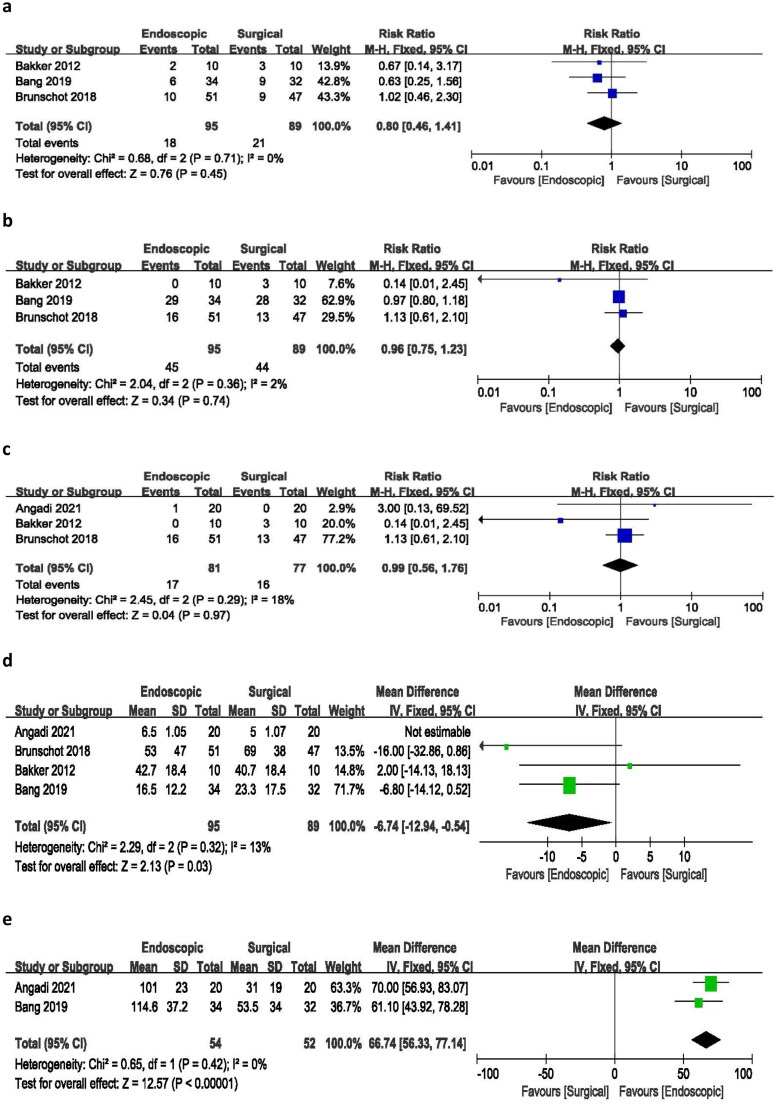
Forest plots for (a) pancreatic endocrine deficiency, (b) pancreatic exocrine deficiency, (c) need for enzyme use, (d) hospital stay, (e) procedure time.

Three studies reported changes in pancreatic endocrine function. The analysis revealed a risk ratio of 0.80 (95% CI 0.46–1.41), with no heterogeneity (I^2^ = 0%) and a *p*-value of 0.45. These findings indicate no significant difference in the incidence of pancreatic endocrine deficiency between endoscopic and surgical approaches (Figure 4(a)).

#### 3.5.10. Pancreatic exocrine deficiency

An analysis of three studies reporting changes in pancreatic exocrine function resulted in a risk ratio of 0.96 (95% CI 0.75–1.23), with minimal heterogeneity (I^2^ = 2%) and a p-value of 0.74. These findings suggest no statistically significant difference between endoscopic and surgical procedures in the incidence of pancreatic exocrine deficiency following treatment (Figure 4(b)).

#### 3.5.11. Need for enzyme use

The analysis of three studies reporting the need for pancreatic enzymes yielded a risk ratio of 0.99 (95% CI 0.56–1.76), with low heterogeneity (I^2^ = 18%) and a *p*-value of 0.97. These results indicate no significant difference in the incidence of needing pancreatic enzymes between the endoscopic and surgical treatment groups (Figure 4(c)).

#### 3.5.12. Hospital stay

Four of the included studies reported on hospital stays. The pooled mean difference was −6.74 days (95% CI −12.94 to −0.54), with minimal heterogeneity (I^2^ = 13%) and a *p*-value of 0.03. The analysis demonstrated that endoscopic treatment was associated with a significantly shorter hospital stay compared to surgical treatment. Notably, the study by Angadi et al. had a significant impact on the overall outcome, and therefore, it was excluded from the hospital stay analysis (Figure 4(d)).

#### 3.5.13. Procedure time

Among the included studies, only two reported the duration of endoscopic and surgical procedures. The pooled mean difference was 66.74 min (95% CI −43.92 to 78.14), with no heterogeneity (I^2^ = 0%) and a *p-*value of 0.000. The analysis indicated that endoscopic treatment required significantly less time to complete compared to the surgical procedure (Figure 4(e)).

## 4. Discussion

In this meta-analysis, we examined the efficacy of endoscopic treatment compared to surgical treatment for necrotizing pancreatitis by analysing data from five RCTs involving a total of 284 treated patients. Our findings revealed that the endoscopic approach yielded better outcomes than the surgical approach in various aspects, including overall major complications, fistula or perforation incidence, hospital stay duration, new onset of organ failure, pancreatic fistula occurrence, surgical site infection rates, and procedure time. These differences were statistically significant.

However, our analysis did not find a statistically significant difference between the two treatment approaches in terms of bleeding incidents, mortality rates, need for additional percutaneous drainage, pancreatic endocrine and exocrine deficiency, and the requirement for pancreatic enzyme use,. Our meta-analysis of the RCT studies has shown different results from the previous studies. Practical and successful management of infected necrotizing pancreatitis (INP) is necessary as the patient population increases. The comparative effectiveness of endoscopic and MIS treatments for INP remains unclear. However, numerous recent studies have primarily focused on endoscopic treatment for INP [25–29]. These studies are limited by small sample sizes and the absence of multi-centre trials.

Medical advancements have resulted in significant progress in the endoscopic treatment of INP. Historically, prior to the twentieth century, conservative management of INP was more prevalent than surgical intervention [6,30]. However, approximately ten years ago, surgical treatment in the form of open necrosectomy gained widespread acceptance as a viable approach for severe pancreatitis [31,32]. In the twenty-first century, with the advent of MIS, there is mounting evidence indicating that minimally invasive approaches are preferable to open surgery [33–37]. Many hospitals now prioritize MIS as the initial treatment option for INP. Recent studies have provided substantial evidence supporting the benefits of endoscopic procedures in the management of infected necrotizing pancreatitis [38–40]. Comparative RCTs evaluating MIS and endoscopic treatments have demonstrated comparable outcomes. A meta-analysis of three studies has shown that an endoscopy-based treatment strategy significantly reduces complications compared to MIS in patients with INP [17]. Our analysis of five RCTs also yielded same results, indicating that endoscopic approaches statistically exhibit fewer major complications than MIS. Another meta-analysis comprising nine studies focusing on INP treatment demonstrated that, in comparison to MIS, endoscopic methods appeared to yield improved short-term outcomes, including reduced pancreatic fistula incidence and decreased hospital stay duration [18]. This meta-analysis corroborated these findings and further indicated that endoscopic procedures outperformed MIS in various aspects. Moreover, an additional meta-analysis involving 190 individuals revealed that endoscopic treatment offered comparable outcomes to surgical approaches with added benefits [19]. However, in this study, incorporated more recent research and produced divergent results compared to previous studies.

A recently published ExTENSION report of RCT with a long-term follow-up of 6 months has also demonstrated that for the treatment of IPN, the endoscopic step-up strategy was not found to be more effective than the surgical step-up technique in reducing the risk of mortality or major complications. However, patients who received the endoscopic method had a significantly lower risk of developing pancreatic fistulas and required fewer reinterventions [41]. In our analysis, studies showing results of initial follow-up have demonstrated that the endoscopic approach performs better than MIS with less major complication, hospital stay, cases of fistula or perforation as well as new-onset of organ failure. Long-term follow-up also stated that the endoscopic approach performs better or the same as the MIS approach. Another study of 2281 patients evaluated the management of infected necrotizing pancreatitis through surgical, endoscopic, or percutaneous approaches. The study found that the endoscopic approach was associated with the lowest risk of inpatient mortality (hazard ratio (HR) 0.27; 95% CI 0.08–0.90; *p* = 0.033), adverse events (*p* < 0.001), length of stay (*p* < 0.001), and total cost [42]. This recent study also confirms the results of our analysis. A meta-analysis revealed that the step-up method is the method that performs substantially better [43]. Many studies have shown that the endoscopic approach using LAMS is the best option for the treatment of WON. As it’s a less invasive and cost-effective option [44,45]. There was a difference in cost of $41,662 between the two approaches, with the endoscopic method costing $75,830 up to 6 months of follow-up and the MIS method costing $117,492 (*p* = 0.039) [15]. From randomization to six months after treatment, the endoscopic step-up strategy cost €60,228 per patient, while the surgical step-up strategy costs €73,883 per patient. The average disparity was €13,655 as a result [16]. Endoscopic treatment of different pancreatobiliary diseases has shown promising results [46–48].

This meta-analysis found that patients who underwent endoscopic therapy had a shorter hospital stay compared to those who underwent MIS (*p* = 03). The reasons for this difference are likely multifactorial. Firstly, surgery has a higher threshold for reintervention following the initial treatment than endoscopy. Secondly, surgical complications, such as pancreatic fistula, may require additional interventions or readmissions. Thirdly, multi-organ failure, which is more common in surgical patients, can lead to long-term morbidity and prolonged hospitalization. The endoscopic method has reduced infection risk and reduced stay, but MIS patients needed long-term care, which affected life quality, risked infection, caused an external pancreatic fistula, and increased expenses. The endoscopic procedure re-introduces pancreatic fluid (PF) into the GI tract, preventing electrolytes and fluid loss compared to the MIS procedure, where PF is drained out of the body. According to the findings of a recent study about the quality of life after INP treatment, endoscopic treatment provides a higher overall quality of life in terms of the patient’s health when compared to the surgical method [9]. The endoscopic technique has been demonstrated to yield the same or comparable results across all of the most recent investigations. The surgery group had a higher incidence of both newly developed and chronic cardiovascular organ failure [16]. Other single organ failure incidents were almost same for the both groups. But there is a need for further investigation with long-term follow-up to support these results.

Our study has several limitations that should be acknowledged. Firstly, the final analysis only comprised five RCTs, and some studies had missing data for certain outcomes. Furthermore, due to variations in follow-up duration and protocols among the included studies, data on recurrence rates and long-term complications were not available for analysis. Another limitation of this meta-analysis is the small sample sizes of the included studies, which may impact the generalizability of the findings. Furthermore, the major complication (sudden loss of function or failure of one or more organs in the body, such as the heart, lungs, or kidneys, leading to death) were not discussed one by one in detail. Additionally, different types of stents were used in the studies, introducing potential variability in the overall results of endoscopic therapy. Although we employed a fixed-effect model to compare the two groups, certain outcomes exhibited heterogeneity, necessitating cautious interpretation of these findings. Moving forward, there is a need for larger-scale RCTs that utilize standardized stent types and treatment approaches to further strengthen the evidence base in this field.

## Conclusion

The findings of this meta-analysis support the preference for endoscopic management over surgical treatment for infected necrotizing pancreatitis (INP) based on its lower complication rate, improved patient quality of life, and lower associated expenses.

## Data Availability

All data generated or analysed during this study are included in this published article.
